# Potential risk factors associated with diabetic mellitus in patients with Febrile upper urinary tract calculi with infection

**DOI:** 10.1371/journal.pone.0325724

**Published:** 2025-06-27

**Authors:** Meng Xu, Shaoqiang Xing, Xuefeng Zhang, Yongchao Jiang, Yun Chen

**Affiliations:** 1 Department of Urology, Yantaishan Hospital, Yantai, Shandong, China; 2 Department of Urology, Tianjin First Central Hospital, Tianjin, China; 3 Department of Urology, Weihai Central Hospital, Weihai, Shandong, China; Memorial Sloan Kettering Cancer Center, UNITED STATES OF AMERICA

## Abstract

**Purpose:**

Patients with febrile upper urinary tract calculi with infection (FUUTCI) are prone to develop or have developed severe infection. This research aimed to evaluate the potential risk factors associated with diabetes mellitus (DM) in patients with FUUTCI.

**Materials and methods:**

From September 2018 to December 2023, patients with FUUTCI admitted to our hospital were retrospectively studied. The patients were divided into a diabetic group (n=52) and a non-diabetic group (n=148), and the differences in demographics, etiology, infection indicators on admission, treatment, and outcome between the two groups were compared. Then regression analysis was performed for gender, stone location, occurrence of urinary sepsis, septic shock, use of pressors, bacterial multiresistance, positive fungal culture, and use of two or more antibiotics.

**Results:**

Compared with non-diabetic patients (148 cases, 74.0%), diabetic patients (52 cases, 26.0%) had a higher prevalence of cardiovascular and cerebrovascular diseases (P=0.031), the rate of using two or more antibiotics (P=0.029), the positive rate of yeast culture (P=0.037), the procalcitonin value of admission or emergency (P=0.022). There was a significant difference in stone location (P=0.039). Regression analysis showed that DM was an independent risk factor for febrile urinary tract infection in patients with kidney stones compared to patients with ureteral stones (P=0.032).

**Conclusions:**

In patients with FUUTCI, the risk factors associated with DM made treatment more complicated. In patients with FUUTCI and under the premise of active treatments, DM was not a risk factor for urosepsis, septic shock, use of vasoactive drugs, and infection of multi-drug resistant bacteria. Compared to patients with ureteral calculi, DM was an independent risk factor for febrile urinary tract infection in patients with kidney calculi.

## Introduction

Patients with upper urinary tract calculi (UUTC) complicated with infection who present with fever symptoms are at risk of developing or have developed urosepsis and septic shock, which can be life-threatening. The main treatment methods currently include emergency decompression, early antibiotic therapy, and close monitoring, and severe cases require intensive care treatment. After infection control, selective endoluminal surgery for stones can be performed [1]. The risk factors for UUTC obstruction complicated by urinary tract infection (UTI) developing into urosepsis and septic shock have not been well understood [2]. Meanwhile, the indications for decompression in what circumstances and when are still unclear, mainly relying on the experience of clinical doctors.

Diabetes mellitus (DM) is considered a significant risk factor for both UTI [3–5] and urinary stone formation [6,7]. Calculi and UTI always coexist and are mutually causal [8]. Patients with urinary calculi are also at increased risk of developing diabetes [9]. Stone obstruction is strongly associated with more severe UTIs. Severe infections can also affect blood sugar levels [10], and the stress hyperglycemia can vary with the change of disease condition. The interplay among these three factors necessitates further investigation. Current clinical research primarily focuses on the overall effects of diabetes or blood sugar control on various UTIs or their implications in postoperative infections following calculi surgery. There is limited literature specifically examining the influence of diabetes on patients with UTIs associated with calculi before calculi lithotripsy operation from a urological perspective. Previous studies have shown that there may be differences in gender, cardiovascular and cerebrovascular disease, pathogens, urosepsis, septic shock, length of hospital stay, and other factors between patients with UUTC with infection with DM and patients with UUTC with infection without DM [2–4,7,11–13]. The differences in these factors have an important impact on the severity, course, and treatment of patients with UUTC with infection. However, the findings of the current researches on these risk factors are inconsistent and controversial. Therefore, this study aims to explore the potential risk factors associated with diabetic mellitus patients with febrile upper urinary tract calculi with infection (FUUTCI), to distinguish the differences between FUUTCI patients with DM and FUUTCI patients without DM, and to provide valuable insights for the clinical diagnosis and treatment of FUUTCI.

## Materials and methods

### Research design

We conducted a retrospective analysis of the clinical data of patients admitted to the Urology department of our medical institution from September 1, 2018, to December 31, 2023. We accessed the data using the electronic medical record system in the second half of February 2024. Inclusion criteria for the study population: patients diagnosed with UUTC accompanied by UTI with fever symptoms after admission or within three days before admission. An axillary body temperature >37°C was considered fever. UTI was defined as a positive urine culture or a comprehensive diagnosis based on clinical symptoms, signs, urine routine, blood routine, and other laboratory tests and imaging findings. The bacterial culture was positive when the bacterial count of midstream urine culture was  ≥ 10^5^ cfu/ml in women,  ≥ 10^4^ cfu/ml in men, or ≥10^4^ cfu/ml in catheterization or renal pelvis urine. The number of yeast colonies in urine culture ≥10^3^ cfu/ml was culture positive. Exclusion criteria for the study population: accompanied by other diseases that may cause fever (such as coronavirus disease 2019, infection of other parts.5 cases), accompanied by other serious diseases other than diabetes (such as life-threatening patients in the late stage of malignant tumor, kidney transplantation, leukemia.2cases), pregnancy (2 cases), postoperative urinary diversion (2 cases), Surgical procedures that may cause fever were performed before fever (such as stone lithotripsy, waiting for reoperation after previous stone lithotripsy, or fever after undergoing ureteral stent placement or nephrostomy), with implant effects (indwelling or long-term indwelling double-J tube or nephrostomy tube before fever.7 cases), use of sodium-dependent glucose transporter 2 (SGLT2) inhibitors before admission (1 case), spontaneous rupture of the renal pelvis or ureter (1 case), unknown diagnosis (1 case), and severe lack of information. A total of 200 patients were included according to these criteria. All patients had undergone urinary CT or ultrasound examination before admission. Upper urinary tract stone obstruction was defined as upper urinary tract stones located in the position causing obstruction, regardless of the hydronephrosis or hydroureter. If the patients were not discharged after infection control and underwent direct surgical treatment, the data for these cases were only collected up to the day before the stone surgery (the length of hospitalization was also truncated at this time). The diagnostic criteria for urosepsis is a rapid increase score of ≥2 points in the Sequential Organ Failure Assessment due to UTI. The diagnostic criteria for septic shock are persistent hypotension in septic patients after full-volume resuscitation, requiring vasoconstrictors to maintain a mean arterial pressure ≥65 mmHg and a serum lactate level >2 mmol/L. Multi-drug resistant bacteria was defined as bacteria resistant to 3 or more types of antibiotics (excluding intermediate antibiotics), excluding natural drug resistance. The patients were divided into a diabetic group and a non-diabetic group according to whether they suffered from diabetes. Patients with diabetes were identified as follows: (1) The patients had been previously diagnosed with diabetes; (2) The patients had not been diagnosed with diabetes before, but were found with elevated blood glucose after admission, which did not return to normal after the stress state was eliminated, and they were subsequently diagnosed as DM by a metabolic physician. The demographic characteristics, etiology, decompression, length of hospital stay, antibiotic use, infection indicators on admission, incidence of urosepsis, incidence of septic shock, admission to intensive care unit (ICU), and use of vasopressors were compared between the two groups. Further univariate and multivariate logistic regression analysis was conducted about the impact of diabetes on gender, stone location, the occurrence of urosepsis, septic shock, use of vasopressors, multiple drug resistance of bacteria, positive fungal culture, and use of two or more antibiotics.

### Statistical methods

The data were analyzed by SPSS 27 software. Except for individual data, our measurement data were all non-normal distribution, so median and range are uniformly used for description. Non-parametric tests such as the Mann-Whitney U test and the Kruskall-Wallis test were used for comparison in groups. Count data are described by the number of cases. Unordered data were compared by chi-square test or Fisher’s exact test, and one-way ordered data were compared by nonparametric test. The statistically significant targets and several other interested targets were selected for univariate logistic regression analysis, and then possible confounding factors were included for multivariate logistic regression analysis. Results are expressed in odds ratio (OR) and adjusted odds ratio (AOR). P < 0.05 was considered statistically significant.

### Ethical statement

This study was approved by the Ethics Review Committee of Weihai Central Hospital (NO.LL-2023–104). We abide by the Helsinki Declaration. All patients were treated according to the guidelines and with informed consent. All patient information was anonymized, authors could not identify individual participants. Because this was a retrospective study, the ethics committee approved an exemption from informed consent.

## Results

### General case data and comparison between groups

A total of 200 patients met the study criteria. There were 67 (33.5%) males and 133 (66.5%) females, with a median age of 65 years (range 21–92). The age group with the most patients was 60 –79 years old, with 113 cases. There were 52 patients (26.0%) with diabetes and 148 patients (74.0%) without diabetes. See Table 1.

**Table 1:** The demographic information and comparison between groups.

	Total(n = 200)	DM(n = 52)	No DM(n = 148)	P
**Gender(%)**				0.131
**Male**	67(33.5)	13(25.0)	54(36.5)	
**Female**	133(66.5)	39(75.0)	94(63.5)	
**Age, year**				0.087
**20-39**	13	1	12	
**40-59**	48	10	38	
**60-79**	113	33	80	
**≥80**	26	8	18	

The median length of hospital stay was 8 days (range 1–48). There were 87 cases (43.5%) of multiple calculi. There were 107 cases (53.5%) of ureteral calculi, 27 cases (13.5%) of renal calculi, and 66 cases (33.0%) of ureteral calculi combined with renal calculi, including staghorn calculi. Of patients with renal calculi, only two were without obstruction. The median of the longest diameter of stones in all patients (using the sum of lengths for patients with multiple stones) was 1.5 cm (range 0.2–16). One hundred and five patients (52.5%) had underlying cardiovascular and cerebrovascular diseases, such as hypertension, cardiac insufficiency, severe arrhythmia, coronary atherosclerosis, myocardial infarction, cerebral thrombosis, and cerebral hemorrhage. Urosepsis occurred in 101 cases (50.5%). Septic shock occurred in 22 cases (11.0%) and 4 cases (2.0%) died. During the treatment process, 12 cases (6.0%) were admitted to the ICU. Vasoactive drugs were used in 31 cases (15.5%), including norepinephrine, m-hydroxylamine, or dopamine, which were given by micro-pump. Forty-nine diabetic patients were treated with oral drugs or insulin injections. During hospitalization, 15 diabetics underwent intravenous drip or micro-pump infusion of insulin dissolved in normal saline, and 22 diabetics required the assistance of metabolic physicians to control blood glucose.

A total of 173 cases (86.5%) underwent nephrostomy or ureteral stent placement for decompression, of which 22 cases (12.7%) successfully underwent nephrostomy, and 151 cases (87.3%) successfully placed double J tubes under cystoscopy or ureteroscopy. Among them, 120 patients (69.4%) underwent emergency decompression on the day of admission or the following day. Twenty-seven patients (13.5%) did not undergo decompression, of which stones spontaneously discharged in 12 patients (6.0%) during hospitalization, one patient underwent extracorporeal shock wave lithotripsy after anti-infection treatment, one patient underwent ureteroscopic lithotripsy directly after anti-infection treatment, two cases were treated with antibiotics combined with lithotripsy, four cases were not decompressed due to no obstruction or mild renal calyx obstruction, seven cases refused decompression treatment. Seven patients (3.5%) underwent percutaneous nephrolithotomy or ureteroscopic surgery directly after the infection was controlled by decompression and antibiotics, and the other patients were asked to return to the hospital for surgery or reexamination 2–4 weeks after discharge.

Among the patients with FUUTCI, there was a significant difference in stone location between diabetic patients and non-diabetic patients, P = 0.039. The prevalence of cardiovascular and cerebrovascular diseases in diabetic patients was higher, P = 0.031. The procalcitonin (PCT) value on admission in diabetic patients was higher, P = 0.022. There were no statistically significant differences between diabetic patients and non-diabetic patients in terms of the longest diameter of stones, multiple stones, length of hospital stay, white blood cell (WBC) on admission, platelet count (PLT) on admission, whether decompression was performed, decompression methods, whether emergency decompression was performed, use of vasoactive drugs, occurrence of urosepsis, septic shock, admission to ICU, and mortality (P > 0.05). See Table 2.

**Table 2:** Clinical factors and comparison between groups.

		Total(n = 200)	DM(n = 52)	No DM(n = 148)	P
**Stone associated obstruction(%)**		198 (98.9)	51 (98.1)	147 (99.3)	0.453
**Multiple stones(%)**		87 (43.5)	26 (50.0)	61 (41.2)	0.272
**Median of the longest stone diameter, cm**		1.5 (0.2–16)^a^	1.7 (0.2–9)^a^	1.5 (0.2–16)^a^	0.112
**Stone location(%)**					0.039
**Ureteral stones**		107 (53.5)	22 (42.3)	85 (57.4)	
**Kidney stones**		27 (13.5)	12 (23.1)	15 (10.1)	
**Kidney stones combined with ureteral stones**		66 (33.0)	18 (34.6)	48 (32.4)	
**Cardiovascular and cerebrovascular diseases(%)**		105 (52.5)	34 (65.4)	71 (48.0)	0.031
**Death(%)**		4 (2.0)	1 (1.9)	3 (2.0)	1.000
**WBC at admission, × 10** ^**9**^ **/L**		11.3(1.98–49)^a^	9.61(2.08–30.14)^a^	11.605(1.98–49)^a^	0.097
**PCT at admission, ng/ml**		17.32(0.029- ﹥ 100)^a^	42.34(0.073- ﹥ 100)^a^	12.23(0.029- ﹥ 100)^a^	0.022
**PLT at admission, × 10** ^**9**^ **/L**		174.5(21–1137)^a^	176(21–385)^a^	174.5(33–1137)^a^	0.710
**Urosepsis(%)**		101 (50.5)	27 (51.9)	74 (50.0)	0.811
**Septic shock(%)**		22 (11.0)	7 (13.5)	15 (10.1)	0.510
**Use of vasoactive drugs(%)**		31 (15.5)	9 (17.3)	22 (14.9)	0.675
**Admission to ICU(%)**		12 (6.0)	6 (11.5)	6 (4.1)	0.083
**Median length of stay(%)**		8 (1–48)^a^	9 (1–34)^a^	7 (2–48)^a^	0.078
**Decompression patients(%)**		173 (86.5)	44 (84.6)	129 (87.2)	0.644
**Decompression methods(%)**					0.403
**Ureteral stent placement**		151 (87.3)^b^	40 (90.9)^b^	111 (86.0)^b^	
**Nephrostomy**		22 (12.7)^b^	4 (9.1)^b^	18 (14.0)^b^	
**Emergency decompression(%)**		120 (69.4)^b^	32 (72.7)^b^	88 (68.2)^b^	0.792
**Reasons for no decompression** **(%)**	**Spontaneous stone passage**	12 (6.0)	1 (1.9)	11 (7.4)	0.191
	**Direct surgery after anti-infection**	2 (1.0)	1 (1.9)	1 (0.7)	
	**Antibiotics, expulsion and dissolution treatment**	2 (1.0)	1 (1.9)	1 (0.7)	
	**Refuse decompression**	7 (3.5)	3 (5.8)	4 (2.7)	0.379
	**No obstruction or mild renal calyx obstruction**	4 (2)	2 (3.8)	2 (1.4)	0.278

^a^ Range.

^b^ Ratio in decompression patients.

ICU: intensive care unit; ESBL: extended-spectrum beta-lactamases; WBC: white blood cell; PCT: procalcitonin; PLT: platelet count; DM: diabetes mellitus.

### Bacterial culture results and comparison between groups

Due to the refusal of individual patients or other reasons, 184 cases (92.0%) underwent bacterial culture, and the positive rate was 51.6% (95 cases). Among them, 177 patients (88.5%) underwent urine culture, 175 patients (87.5%) underwent midstream urine culture, 22 patients (11.0%) underwent renal pelvis urine culture, and 79 patients (44.4%) were positive for urine culture. Blood bacterial culture was performed in 81 patients (40.5%), and 38 patients (46.9%) were positive. Among the culture-positive patients, *Escherichia coli* (67.8%) was the most common bacteria, followed by *Klebsiella pneumoniae* (14.9%), *Proteus mirabilis* (9.2%), *Enterococcus faecium* (5.7%), and *Enterococcus faecalis* (4.6%). Yeast was isolated from 14 patients (14.7%), all of whom were treated with two or more antibiotics. Multidrug-resistant bacteria were isolated from 62 patients (71.3%), and extended-spectrum β-lactamase (ESBL) was positive in 39 patients (44.8%). Five patients cultured two types of bacteria, five patients cultured yeast and one type of bacteria, and one patient cultured yeast and two types of bacteria.

In patients with positive blood or urine culture, the yeast infection rate in the diabetic group was higher than that in the non-diabetic group, P = 0.037. There were no significant differences in culture method, positive rate, bacterial species, drug resistance, and other pathogenic characteristics. See Table 3.

**Table 3:** Bacterial culture results and comparison between diabetics and non-diabetics.

	Total(n = 200)	DM(n = 52)	No DM(n = 148)	P
**Patients participated in culture(%)**	184 (92.0)	48 (92.3)	136 (91.9)	0.924
**Pelvis or midstream urine(%)**	177 (88.5)	47 (90.4)	130 (87.8)	0.620
**Positive (positive rate of urine culture,%)**	79 (44.4)	18 (38.3)	61 (46.6)	0.328
**Blood culture(%)**	81 (40.5)	21 (40.4)	60 (40.5)	0.984
**Positive (positive rate of blood culture,%)**	38 (46.9)	12 (57.1)	26 (43.3)	0.275
**Patients with positive culture(%)**	95 (51.6)^a^	23 (47.9)^a^	72 (52.9)^a^	0.549
**Yeast(%)**	14 (14.7)^b^	7 (30.4)^b^	7 (9.7)^b^	0.037
**Patients with positive bacterial culture(%)**	87 (47.3)^a^	18 (37.5)^a^	69 (50.7)^a^	0.114
***Escherichia coli*(%)**	59 (67.8)^c^	12 (66.7)^c^	47 (68.1)^c^	0.907
***Klebsiella pneumoniae*(%)**	13 (14.9)^c^	2 (11.1)^c^	11 (15.9)^c^	1.000
***Proteus mirabilis*(%)**	8 (9.2)^c^	1 (5.6)^c^	7 (10.1)^c^	1.000
***Enterococcus faecium*(%)**	5 (5.7)^c^	2 (11.1)^c^	3 (4.3)^c^	0.275
***Enterococcus faecalis*(%)**	4 (4.6)^c^	1 (5.6)^c^	3 (4.3)^c^	1.000
***Pseudomonas aeruginosa*(%)**	1 (1.1)^c^		1 (1.4)^c^	
***Enterobacter aerogenes*(%)**	1 (1.1)^c^		1 (1.4)^c^	
***Acinetobacter baumannii*(%)**	1 (1.1)^c^		1 (1.4)^c^	
***Staphylococcus aureus*(%)**	1 (1.1)^c^		1 (1.4)^c^	
***Human Staphylococcus*(%)**	1 (1.1)^c^	1 (5.6)^c^		
**Gram staining classification(%)**				0.222
**Gram-positive cocci**	9 (10.3)^c^	3 (16.7)^c^	6 (8.7)^c^	
**Gram-negative rods**	76 (87.4)^c^	14 (77.8)^c^	62 (89.9)^c^	
**Gram-positive cocci + Gram-negative rods**	2 (2.3)^c^	1 (5.6)^c^	1 (1.4)^c^	
**Patients with multidrug-resistant bacteria(%)**	62 (71.3)^c^	11 (61.1)^c^	51 (73.9)^c^	0.285
**ESBL positive patients(%)**	39 (44.8)^c^	5 (27.8)^c^	34 (49.3)^c^	0.102
**Patients with two or more pathogens(%)**	11(12.6)^c^	3(16.7)^c^	8(11.6)^c^	0.690

^a^ Percentage in participants in culture.

^b^ Percentage in culture-positive cases.

^c^ Percentage in positive bacterial culture cases.

ESBL: extended-spectrum β-lactamase; DM: diabetes mellitus.

### The use of antibiotics and comparison between groups

During hospitalization, 99.5% of the patients were treated with antibiotics, all of whom received intravenous antibiotics, and only 1 patient received oral antibiotics together. Among them, 112 cases (56.3%) were treated continuously or simultaneously with two or more antibiotics. The most commonly used antibiotic was Piperacillin Tazobactam, followed by Etimicin, Levofloxacin, and Imipenem-cilastatin Sodium. Broad-spectrum penicillins were used in 155 cases (77.9%), aminoglycosides in 70 cases (35.2%), carbapenems in 46 cases (23.1%), quinolones in 39 cases (19.6%), cephalosporins in 33 cases (16.6%), nitroimidazole in 7 cases (3.5%). There were no significant differences in the types of antibiotics used between diabetic and nondiabetic patients. Diabetics used more kinds of antibiotics, P = 0.044. More diabetic patients used 2 or more antibiotics, P = 0.029. The main reasons for using two or more antibiotics included simultaneous combined applications (81 cases, 72.3%), upgrading or downgrading adjustment of antibiotics (41 cases, 36.6%), empirical replacement of antibiotics of equal strength (14 cases, 12.5%), and adjusting antibiotics due to drug resistance (9 cases, 8.0%). There were no significant differences in the reasons between the two groups. There was also no significant difference in the use of more complex antibiotic regimens such as simultaneous combined applications plus other reasons (P = 0.640). See Table 4.

**Table 4:** The use of antibiotics and comparison between diabetics and non-diabetics.

	Total(n = 199)	DM(n = 52)	No DM(n = 147)	P
**Broad-spectrum penicillins(%)**	155 (77.9)	38 (73.1)	117 (79.6)	0.331
**Piperacillin sodium and tazobactam sodium**	150 (75.4)	38 (73.1)	112 (76.2)	
**Mezlocillin sodium and sulbactam sodium**	9 (4.5)	2 (3.8)	7 (4.8)	
**Aminoglycosides(%)**	70 (35.2)	19 (36.5)	51 (34.7)	0.811
**Etimicin**	69 (34.7)	19 (36.5)	50 (34.0)	
**Amikacin**	1 (0.5)		1 (0.7)	
**Carbopenems(%)**	46 (23.1)	16 (30.8)	30 (20.4)	0.128
**Imipenem and cilastatin sodium**	25 (12.6)	9 (17.3)	16 (10.9)	
**Meropenem**	23 (11.6)	8 (15.4)	15 (10.2)	
**Quinolones(%)**	39 (19.6)	13 (25.0)	26 (17.7)	0.254
**Levofloxacin**	38 (19.1)	12 (23.1)	26 (17.7)	
**Moxifloxacin**	1 (0.5)	1 (1.9)		
**Cephalosporins(%)**	33 (16.6)	10 (19.2)	23 (15.6)	0.550
**Cefoperazone Sodium and Sulbactam Sodium**	21 (10.6)	5 (9.6)	16 (10.9)	
**Ceftazidime**	9 (4.5)	4 (7.7)	5 (3.4)	
**Ceftriaxone**	1 (0.5)		1 (0.7)	
**Cefdinir**	1 (0.5)	1 (1.9)		
**Nitroimidazoles(%)**	7 (3.5)	1 (1.9)	6 (4.1)	0.679
**Metronidazole**	2 (1.0)		2 (1.4)	
**Tinidazole**	5 (2.5)	1 (1.9)	4 (2.7)	
**Cefminox**	2 (1.0)		2 (1.4)	
**Linezolid**	1 (0.5)	1 (1.9)		
**Vancomycin**	2 (1.0)		2 (1.4)	
**Tigecycline**	1 (0.5)	1 (1.9)		
**Number of antibiotics used(%)**				0.044
**1 kind**	87 (43.7)	16 (30.8)	71 (48.3)	
**2 kinds**	78 (39.2)	25 (48.1)	53 (36.1)	
**3 kinds**	20 (10.1)	7 (13.5)	13 (8.8)	
**4 or more kinds**	14 (7.0)	4 (7.7)	10 (6.8)	
**Use of 2 or more antibiotics(%)**	112 (56.3)	36 (69.2)	76 (51.7)	0.029
**Reasons for using 2 or more antibiotics(%)**				
**Simultaneous combined applications**	81 (72.3)^a^	25 (69.4)^a^	56 (73.7)^a^	0.640
**Involving drug resistance**	9 (8.0)^a^	1 (2.8)^a^	8 (10.5)^a^	0.267
**Upgrades and downgrades**	41 (36.6)^a^	15 (41.7)^a^	26 (34.2)^a^	0.444
**Empirical replacement of the same strength**	14 (12.5)^a^	5 (13.9)^a^	9 (11.8)^a^	1.000
**Simultaneous combined applications + other reasons**	28 (25.0)^a^	10 (27.8)^a^	18 (23.7)^a^	0.640

^a^Proportion in use of 2 or more antibiotics.

DM: diabetes mellitus.

### Regression analysis of DM as an influencing factor

Univariate and multivariate logistic regression were performed to investigate the influence of DM on some potential factors. Some possible confounding factors were selected according to the actual significance and statistical association during the multivariate logistic regression analysis. The details were as follows: when we studied the impact on patients of different genders, factors such as age, cardiovascular and cerebrovascular diseases, decompression methods, and the use of two or more antibiotics were included; When studying the impact on patients with different stone locations, factors such as the longest stone diameter, WBC and PLT on admission, decompression methods, emergency decompression, septic shock, and the use of two or more antibiotics were included; When studying the impact on urosepsis, septic shock, and the use of vasoactive drugs, factors such as age, location of stones, PLT on admission, length of hospital stay, emergency decompression, positive culture, and use of two or more antibiotics were included; Factors such as gender, age, length of hospital stay, WBC and PLT on admission, septic shock, emergency decompression, and use of two or more antibiotics were included to study the effects on the multiple drug resistance of bacteria; Gender, age, length of hospital stay, WBC and PLT on admission, admission to ICU, cardiovascular and cerebrovascular diseases, and use of two or more antibiotics were involved to study the effects on positive yeast culture; We included gender, age, admission WBC and PLT, length of hospital stay, urosepsis, and positive culture to research the effects on using two or more antibiotics.

Univariate regression analysis showed that DM was a risk factor for the use of two or more antibiotics (OR 2.132 [1.089–4.171], P = 0.027), and yeast infection (OR 3.146 [1.042–9.500], P = 0.042), however, multivariate analysis showed it was not an independent risk factor. Compared with patients with ureteral calculi, DM increased the risk of UTI with fever symptoms in patients with renal calculi (OR 3.091 [1.267–7.543], P = 0.013), which was an independent risk factor (AOR 4.382 [1.132–16.958], P = 0.032). DM was not a risk factor for gender, progression to urosepsis, septic shock, use of vasoactive drugs, or infection of multi-drug resistant bacteria in patients with FUUTCI. See Table 5.

**Table 5:** Univariate and multivariate regression analysis.

		Univariate			Multivariate		
		OR	95%CI	P	AOR	95%CI	P
**Gender (female)**		1.723	0.846-3.510	0.134	1.526	0.708-3.289	0.281
**Stone location (compared with ureteral stones)**	**Kidney stones**	3.091	1.267-7.543	0.013	4.382	1.132-16.958	0.032
	**Kidney stones combined with ureteral stones**	1.449	0.708-2.966	0.310	2.229	0.650-7.641	0.202
**Urosepsis**		1.080	0.574-2.032	0.811	0.994	0.460-2.150	0.989
**Septic shock**		1.379	0.529-3.598	0.511	0.930	0.242-3.579	0.916
**Use of vasoactive drugs**		1.199	0.513-2.803	0.676	0.911	0.297-2.798	0.871
**Multidrug resistance of bacteria**		0.555	0.187-1.649	0.289	0.481	0.143-1.623	0.238
**Yeast**		3.146	1.042-9.500	0.042	1.859	0.446-7.750	0.395
**Use of 2 or more antibiotics**		2.132	1.089-4.171	0.027	2.080	0.923-4.689	0.077

AOR: adjusted odds ratio; OR: odds ratio.

## Discussion

Fever is a systemic manifestation of UTI, which proves that the infection has developed to a certain degree. In some elderly and weak patients, the body temperature may not significantly rise when infection occurs. Previous studies often defined febrile UTI as body temperature ≥38°C [11,14]. These studies did not explain the method of body temperature measurement, and some febrile patients may not be included, which could not reflect the full picture of febrile patients. Therefore, we considered the axillary temperature >37°C as the criterion and included all patients exhibiting fever symptoms in the study.

A study has shown that DM further increases the morbidity of upper urinary calculi in women with UTI [7]. Several other studies have shown that diabetes increased UTI risk within men and women subgroups by a similar magnitude [3]. In our study, female patients (66.3%) were 1.97 times that of male patients, which is consistent with the fact that there are more females than males with UTI and contrary to the fact that there are more males than females with calculi. The influence of diabetes on the risk of FUUTCI was similar between different genders.

Urinary calculi are associated with an increased risk of cardiovascular and cerebrovascular diseases [15,16]. UTI also increases the risk [17], but few relevant studies exist. The prevalence of cardiovascular and cerebrovascular diseases in the study population was high. In addition to its high prevalence in the general population, the reason may be related to calculi and UTI. In our study, patients with DM were more likely to suffer from cardiovascular and cerebrovascular diseases, which makes the condition more complicated or more severe [12].

Obstructing UUTC with UTI need decompression, and delayed decompression increases mortality [1,18]. Yoshimura et al [19] found diabetes was not a risk factor for emergency decompression. In our research, no differences in decompression were found in diabetic patients. We found that not all patients with FUUTCI need emergency decompression, and in a few patients, the stones could pass spontaneously after anti-infection and medical expulsive therapy.

In terms of stone location, Lee et al [2] studied patients who underwent emergency ureteral stent placement for ureteral calculi complicated with infection and found no significant difference in the location of ureteral calculi between diabetic and nondiabetic patients. However, there are few directly related studies for reference. In our study, compared with ureteral stones, DM significantly increased the risk of UTI with fever in patients with kidney stones, even after including seven confounding factors. However, there was no difference in the effect between patients with ureteral stones and those with kidney stones combined with ureteral stones. Although in diabetic patients, there were still more ureteral calculi patients than kidney calculi patients who suffered from febrile UTI, DM increased the proportion of renal calculi patients and decreased the proportion of ureteral calculi patients. Ureteral stones generally originate from kidney stones entering the ureter. Ureteral calculi obstruction is associated with more serious infections such as urosepsis, septic shock, and death from urosepsis [20–22]. In our study, 92.6% of patients with renal stones exhibited obstruction, but febrile UTIs in non-diabetic patients may depend more on the obstruction caused by stones entering the ureter. Therefore, it can be inferred that DM may advance the occurrence time of febrile UTIs in patients with calculi, which requires further research.

Currently, the disparity in pathogens between diabetic and non-diabetic patients remains controversial. A systematic review has shown that the species profile of UTI-causing pathogens from diabetic individuals is not different from that in non-diabetics, although diabetes significantly increases the risk of urinary colonization by drug-resistant uropathogenic bacteria [3]. Nevertheless, research by Mirza Sain et al. from Saudi Arabia found no significant differences in the bacterial profile and susceptibility pattern between diabetic and non-diabetic patients [23]. Kobayashi et al. found that among patients with febrile UTI, patients with diabetes or hyperglycemia were liable to have non-*E. coli* uropathogens, and suggested that inconsistent research results regarding the relationship between diabetic status and clinical course may partly come from the difference in uropathogens [11]. Microbial etiology is greatly affected by different regions and populations and may change over time. In our study, *Escherichia coli* occupied an absolute advantage, followed by *Klebsiella pneumoniae*, *Proteus*, *Enterococcus faecalis*, and *Enterococcus faecium*, which was similar to a study on patients with urolith obstructive urosepsis from southern China [24] but differ from the study of Kobayashi et al [11] and another study conducted on patients of urinary calculi with UTI in northern China [25]. Our study had a high proportion of multi-drug resistant bacteria and ESBL-positive bacteria. This makes it necessary to choose β-lactamase-resistant antibiotics for empirical treatment, covering both Gram-negative bacilli and Gram-positive cocci. Although not supported by the multivariate regression analysis, the positive rate of yeast was significantly higher in the diabetic group, which is in line with the study by Mirza Sain et al [23]. Our study did not find differences in culture-positive rate, bacterial species, multi-drug resistant bacteria, and ESBL positivity. A study showed that urosepsis caused by ESBL-positive bacteria infection was associated with longer antibiotic use and hospital stay [26]. Therefore, the inference of Kobayashi et al [11] is reasonable. In our study, patients with DM used more kinds of antibiotics. This means, they received more active and complex antibiotic treatment. However, there is no significant difference in the reasons for using two or more antibiotics, and drug resistance is not the main reason. It was more of an empirical application based on clinical practice, and whether there was a suspicion of antibiotic overtreatment remains to be discussed. The increased yeast infections in diabetic patients may be associated with this.

DM is often considered to be associated with more severe UTI and a more complex course of disease [3,5,13], which can prolong the length of hospital stay [11] and increase the risk of progression to urosepsis and septic shock [4,13,20]. However, A multi-center prospective cohort study showed that DM was not independently associated with a complex course in unselected patients with febrile UTI, and there was no significant difference in the length of hospital stay [12]. Specifically, in patients with stones, several studies have shown that DM is not an independent risk factor for septic shock related to stone obstruction [24,27,28]. A study also found that DM was not an independent risk factor for ureteral calculi associated with urosepsis [29]. Another study by Lee et al showed that DM was not a risk factor for septic shock and ICU admission, and there was no statistical difference in the length of hospital stay and the use of vasoactive drugs [2]. These negative results are consistent with our research. Although all of these studies, including this one, are about the impact of DM on UTI, in different studies, the not identical research objects, the differences in microbial etiology, the more active use of antibiotics, stone location, and other factors may lead to the differences in different research results in many aspects, such as the length of hospital stay and infection outcome. Our study found that patients with diabetes had higher PCT values on admission or in the emergency department, which may mean that patients were more severely infected on admission. Although on the premise of active treatment, DM does not increase the risk of FUUTCI progressing to urosepsis and septic shock, diabetic patients are more likely to develop UTI [3], which increases the patient base. The hospitalization time of diabetic patients is not prolonged, but the higher PCT on admission, more treatment for cardiovascular and cerebrovascular diseases, more complex antibiotic regimens, the need to regulate blood glucose, and the need for micro-pumps to pump insulin in some patients all lead to more complex treatment.

The limitations of this study are that it is a single-center retrospective study, and the sample size is small. It needs to be further confirmed by multi-center prospective research with larger samples. Pathogenic bacteria analysis was limited to the level of patient cases, and a detailed analysis of antibiotic-resistant varieties by strain was not conducted. We did not analyse the impacts of blood glucose control levels before admission. Due to the significant missing data of PCT values, it was not included in the regression analysis of the effects on other factors to ensure the accuracy of the results. Although short-term use of SGLT2 inhibitors in two patients after admission may have interfered with the study results, we tried to recalculate the results after excluding these two patients and found that they did not affect the conclusion. The subjects of this study were only patients with FUUTCI, and the comparison was only made internally, not compared to patients with calculi without infection or people without FUUTCI and DM. Therefore, the applicability of the conclusion is limited.

## Conclusion

In patients with FUUTCI, the risk factors associated with DM makes the treatment more complicated. In patients with FUUTCI and on the premise of active treatment, DM does not prolong the length of hospital stay, and it is not a risk factor for urosepsis, septic shock, the use of vasoactive drugs, and the infection of multi-drug resistant bacteria. Compared to patients with ureteral calculi, DM increases the risk of febrile UTI in patients with kidney calculi, which is an independent risk factor.

## Supporting information

S1 Raw Data. (XLS)
